# Decreased Stroke Presentation Rates at a Comprehensive Stroke Center during COVID-19

**DOI:** 10.1017/cjn.2020.193

**Published:** 2020-09-03

**Authors:** Dar Dowlatshahi, Grant Stotts, Aline Bourgoin, Sophia Gocan, Laura Dunn, Jodi Powell, Frank L. Silver, Greg Walker, Vignan Yogendrakumar, Robert Fahed, Dylan Blacquiere, Michel Shamy

**Affiliations:** Department of Medicine (Neurology), University of Ottawa Brain & Mind Institute and Ottawa Hospital Research Institute, Ottawa, Ontario, Canada; Ottawa Hospital and Champlain Regional Stroke Network, Ottawa, Ontario, Canada; Department of Medicine (Neurology), University of Toronto, and Stroke Program, University Health Network, Toronto, Ontario, Canada; Division of Neurology, Royal Columbian Hospital, University of British Columbia, Vancouver, BC, Canada

**Keywords:** COVID-19, Incidence, Stroke

## Abstract

We reviewed stroke care delivery during the COVID-19 pandemic at our stroke center and provincial telestroke system. We counted referrals to our prevention clinic, code strokes, thrombolysis, endovascular thrombectomies, and activations of a provincial telestroke system from February to April of 2017–2020. In April 2020, there was 28% reduction in prevention clinic referrals, 32% reduction in code strokes, and 26% reduction in telestroke activations compared to prior years. Thrombolysis and endovascular thrombectomy rates remained constant. Fewer patients received stroke services across the spectrum from prevention, acute care to telestroke care in Ontario, Canada, during the COVID-19 pandemic.

## RÉSUMÉ

**Diminution du nombre de patients victimes d’un AVC s’étant présentés dans un centre de soins complets au cours de la pandémie de COVID-19.** C’est dans le contexte sanitaire actuel que nous nous sommes penchés sur la prestation de soins offerts à des patients victimes d’un AVC s’étant présentés dans notre centre de soins de l’AVC et ayant bénéficié également du programme provincial TéléAVC. De février à avril 2017, 2018, 2019 et 2020, nous avons ainsi dénombré le nombre de demandes d’orientation destinées à notre clinique de prévention, d’interventions rapides face à des cas d’AVC (*code strokes*), de thrombolyses, de thrombectomies endovasculaires (TE) et de demandes de consultation au moyen de TéléAVC. Pour le mois d’avril 2020, nous avons observé une réduction de 28 % des demandes d’orientation par rapport au même mois des années antérieures. Dans le cas des interventions rapides et des demandes de consultation au moyen de TéléAVC, cette réduction a été respectivement de 32 et 26 % pour la même période. Les taux de thrombolyses et de TE sont par ailleurs restés les mêmes. Si l’on tient compte de tout l’éventail des soins offerts aux patients, des soins de courte durée aux soins en télémédecine, soulignons enfin que moins de patients ont bénéficié de soins de l’AVC au cours de la pandémie de COVID-19.

Reports from several comprehensive stroke centers have suggested a decline in patient presentations to stroke prevention clinics and to acute stroke services since the initiation of lockdowns during the COVID-19 pandemic.^1,2^ It is possible that fears of COVID-19 exposure have led patients to avoid assessment for transient ischemic attack (TIA) and minor stroke. However, fears of exposure to infection are less likely to prevent patients with severe stroke to present for thrombolysis or endovascular thrombectomy (EVT).

The Ottawa Hospital (Ottawa, Ontario, Canada) is a comprehensive stroke center servicing Canada’s National Capital Region, with a catchment area of 1.2 million. In 2019, 850 patients were admitted with a diagnosis of stroke, of whom 164 received thrombolysis and 105 received EVT. Code Stroke protocols are activated at the emergency department upon presentation of patients with sudden onset of neurological deficits. The Ottawa stroke prevention clinic (SPC) is a rapid access clinic located inside the Ottawa Hospital servicing the national capital region with an average of 2600 new referrals per year.^3^ Acute stroke care at remote hospitals throughout the province of Ontario (population 14 million) is supported by the Ontario telestroke program (OTP).^4^ Currently, there are 31 hospitals that refer to the OTP, including designated district stroke centers (primary stroke centers) and nondesignated hospitals that provide thrombolytic therapy. Emergency physicians at these referring hospitals request a telestroke consultation by calling CritiCall, a provincial program that supports access to emergent care. Connectivity, including 2-way videoconferencing and access to imaging, is supported by the Ontario telemedicine network (OTN). Telestroke neurologists are consulted for patients presenting to OTN referring sites with a suspected acute ischemic stroke within 6 hours of stroke onset or within 12 hours with suspected large vessel occlusion.

Our objective was to perform a rapid review of stroke care delivery at our comprehensive stroke center and through the provincial telestroke system, both before and after the pandemic “shutdown.” We hypothesized that referrals to stroke prevention clinic, code stroke activations, and telestroke activations have declined during the pandemic shutdown.

As part of an ongoing quality assurance program, the Ottawa stroke program and Champlain regional stroke program prospectively collect local data on referrals to the SPC, code stroke presentations, thrombolysis, and EVT. We obtained local research ethics board approval to access this data. CritiCall similarly collects data arounds the number of telestroke activations for ongoing quality assurance; we requested a summary of activations over the timeframe of interest from the Ontario telehealth network, who maintains this database.

We considered mid-March 2020 to represent the start of the COVID-19 pandemic in Ottawa based on several factors: the first confirmed case of COVID-19 in Ottawa on March 11, the official recommendation by the Prime Minister of Canada on March 13 to avoid travel, the declaration of a state of emergency in Ontario (s7.0.1(1) of the Emergency Management and Civil Protection Act) on March 17, and the extension of shutdowns with the City of Ottawa declaring a state of emergency on March 25. We collected data regarding the number of code stroke activations, thrombolysis, EVT procedures, and referrals to the SPC in February, March, and April 2020. We collected similar data from the years 2017 to 2019 as comparators. Event rates, pre- and postshutdown within 2020 and against 2019, levels were compared using single- and multi-interrupted time series analysis, respectively. Statistical analysis was performed using SAS v9.4 (SAS Institute Inc, Cary, NC).

**Figure 1:**
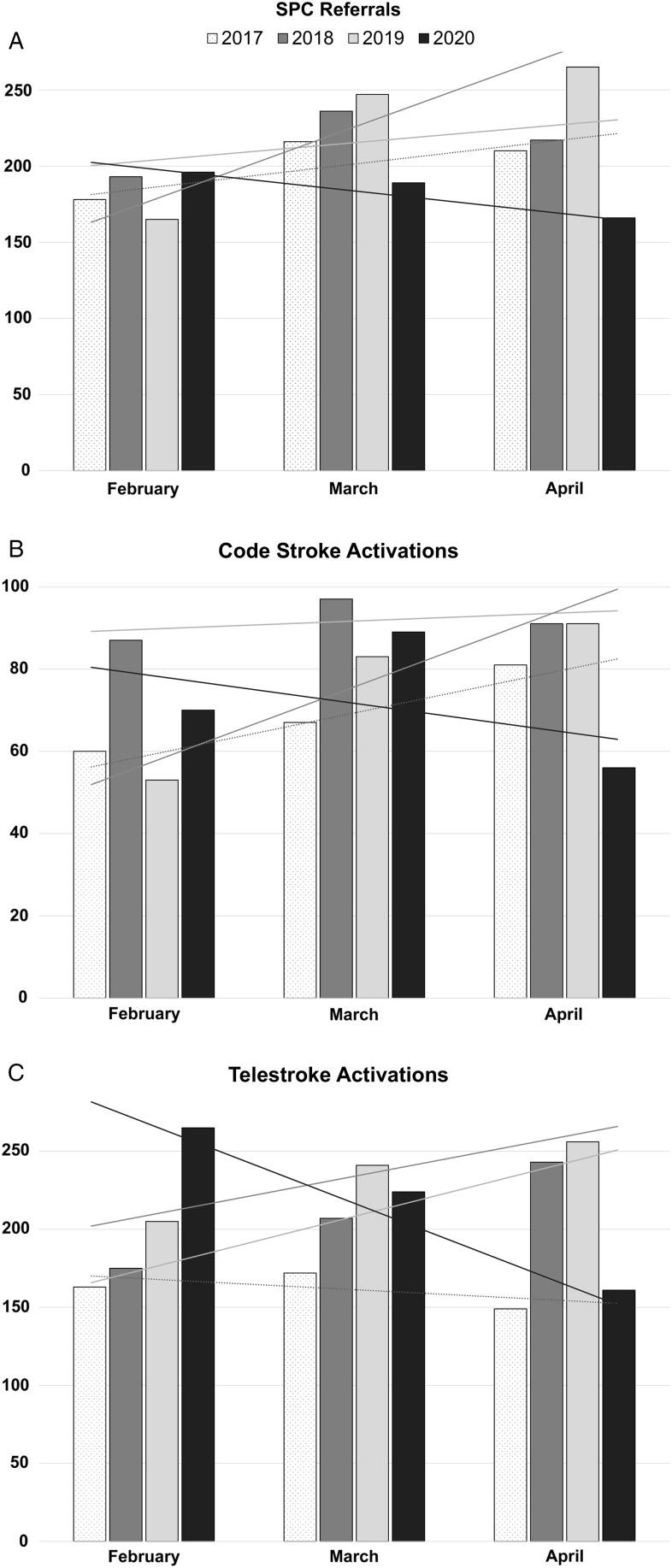
Number of (A) stroke prevention clinic referrals, (B) code stroke activations at the Ottawa Hospital, and (C) Ontario telestroke network activations in 2017–2020 across February, March, and April. Linear trendlines are shown. SPC = stroke prevention clinic.

Events rates shown in Table 1 demonstrate a trend towards decreased SPC referrals, code stroke activations, and telestroke activations in April 2020, following the COVID-19 shutdown. Trends for prior years are demonstrated in Figure 1, which differ from 2020. Administration of tPA or performance of EVT procedures do not follow the same trend towards decrease. As March 2020 Ottawa Hospital code stroke was the only data point with an increase in events, we separated the month into two halves (March 1–15 and 15–31) for further inspection. In the first half of March 2020 (prior to the shutdown), there were 56 code strokes as compared to the second half, where there were 33.

**Table 1:** Rates of stroke prevention clinic referrals, code strokes, thrombolysis, and endovascular thrombectomies at the Ottawa Hospital and telestroke activations in Ontario, Canada from 2017 to 2020 across February, March, and April. SPC = stroke prevention clinic

	February	March	April
**SPC referrals**			
2017	178	216	210
2018	193	236	217
2019	165	247	265
2020	196	189	166
**Code strokes**			
2017	60	67	81
2018	87	97	91
2019	53	83	91
2020	70	89	56
**Code strokes with thrombolysis**			
2017	14	20	16
2018	24	17	22
2019	11	21	23
2020	13	23	19
**Endovascular thrombectomy**			
2017	3	13	10
2018	10	12	15
2019	8	16	15
2020	11	17	11
**Telestroke activations (Ontario wide)**			
2017	163	172	149
2018	175	*207*	243
2019	205	*241*	256
2020	265	224	161

Following the Covid-19 shutdown, stroke code activations from March 15, 2020 to May 15, 2020 decreased significantly compared to 2019 activation levels within that same timeframe (p = 0.03). Event rate comparisons within 2020, pre and postshutdown, demonstrate a trend towards decreased code activations (p = 0.09). Stroke code referrals were not observed to significantly differ pre- and postshutdown (p = 0.36); however, a comparison of 2019 referrals suggest a statistical trend towards reduction in referrals (p = 0.06).

The number of referrals to our SPC has trended downwards by approximately 28% during the month of April 2020 when compared to the average from April 2017 to 2019. Moreover, the number of stroke code activations at our comprehensive stroke center has significantly declined by 31.5% with a relative preservation of the number of administrations of tPA and performance of EVT. A similar pattern is also seen across our provincial telestroke system which had a 25.5% drop in activations in April 2020 compared to the same month in 2017–2019. These findings are consistent with those recently reported by two North American comprehensive stroke centers.^1,2^

We suspect that these findings reflect a reluctance of patients to present to hospital, either in the outpatient setting or through the emergency department, due to fear of contracting COVID-19. This phenomenon was specifically anticipated by a recent statement by the Heart and Stroke Foundation of Canada, which issued a specific best practice recommendation on stroke care during the COVID-19 pandemic.^5^ Our study provides early data to suggest that concerns surrounding acute stroke care provision are indeed warranted.

We emphasize the importance and impact of acute stroke care and stroke prevention, in particular given our finding that the administration of tPA and EVT has not dropped. This suggests that severe strokes are still happening, and it is perhaps patients with milder presentations who are avoiding activation of emergency systems. This is of concern as minor strokes and TIAs are still associated with significant disability^6^ and provide an opportunity for critical preventative interventions.

Our study has several limitations. First, we report results from a single center, albeit a comprehensive stroke center that provides acute stroke care in a large region. However, we support the generalizability of our findings by also demonstrating a similar pattern across a large 31-center telestroke network. Second, we are drawing conclusions from a very short timeframe, which precludes detailed statistical analyses, and therefore we cannot rule out variations unrelated to the COVID-19 pandemic. Third, we were also unable to obtain detailed data from the CritiCall network to perform formal statistical analysis as granular data is only available in a delayed fashion. Nevertheless, we note a consistency of the trends reported across all three domains of stroke care, namely prevention, acute care, and telestroke care, suggesting a true effect. Finally, we do not have access to data showing diagnostic delays, medical errors, or adverse outcomes due to the COVID-19 shutdown.

We cannot conclude that the drop in presentation rates is solely due to patients avoiding hospitals; it is possible that healthcare workers, including emergency physicians and primary care providers, are contributing to the decreased rates of referrals and activations. It is therefore critical to continue to alert clinicians and patients alike that acute stroke treatment and stroke prevention are highly effective, with some of the lowest numbers needed to treat across the field of medicine.^7–9^ It is imperative that COVID-19 does not further disrupt the care of patients experiencing stroke in North America and around the world, particularly if there is a “second wave.” The stroke community must continue to mobilize data to inform governmental agencies, hospital administrators, patient advocacy groups, and the public: stroke care is still necessary and accessible during COVID-19.
